# Can Pursuing Perfection Ignite Sparks of Creativity? How and When Self-Oriented Perfectionism Leads to Radical and Incremental Creativity

**DOI:** 10.3390/bs16091650

**Published:** 2026-09-14

**Authors:** Qing Zhang, Haibo Yu, Wendi Jiang, Shanghao Song, Rui Xiong, Xiaolin Ge

**Affiliations:** 1 School of Economics & Management, Harbin Normal University, Harbin 150025, China; zhangqingat301@mail.bnu.edu.cn; 2 School of Government, Beijing Normal University, Beijing 100875, China; yuhb@bnu.edu.cn; 3 Business School, Beijing Normal University, Beijing 100875, China; wendijiang@mail.bnu.edu.cn; 4 School of Labor Economics, Capital University of Economics and Business, Beijing 100070, China; 5 Collaborative Innovation Center of Assessment Toward Basic Education Quality, Beijing Normal University, Beijing 100875, China; ruixiong@mail.bnu.edu.cn; 6 School of Economics & Management, Beijing Forestry University, Beijing 100083, China; gexiaolin@mail.bnu.edu.cn

**Keywords:** self-oriented perfectionism, job crafting, radical and incremental creativity, work stress

## Abstract

Although perfectionism has received considerable attention from organizational scholars, research on how it enhances employee creativity remains limited. Drawing on the model of proactive motivation and trait activation theory, this study investigates the relationship between employees’ self-oriented perfectionism and creativity (including both radical and incremental creativity), with job crafting as a mediating mechanism. In addition, we incorporate work stress as a moderator and propose a moderated mediation model. Multi-wave survey data were collected from employees in several Chinese organizations. The results show that self-oriented perfectionism is positively associated with both radical and incremental creativity, and that job crafting mediates these relationships. Furthermore, work stress moderates the mediated relationships, such that the positive indirect associations are stronger when work stress is higher. Theoretical and practical implications are discussed.

## 1. Introduction

In the digital era, the economic and social environment is fiercely competitive and volatile, with technology reshaping organizational structures and market landscapes. This compels organizations not only to enhance efficiency and performance, but also to possess robust innovation capabilities in order to sustain competitive advantage and achieve continuous growth (Anderson et al., 2014). To realize organizational innovation and development, employees, who serve as practitioners of daily organizational operations and direct drivers of organizational innovation, possess creative problem-solving abilities that are critical for enhancing organizational effectiveness and resilience in the digital age. By actively exercising creativity in their work, they can generate novel ideas to help achieve organizational goals (Shalley, 1995; Zhou & Shalley, 2024). Creativity has been defined as “the development of novel, potentially useful ideas” (Shalley et al., 2004, p. 934). Given the multidimensional and complex nature of creativity, the literature has distinguished between radical creativity and incremental creativity, each of which plays important roles in different work processes and stages (Bulut et al., 2022; Madjar et al., 2011; Ren & Song, 2024). Radical creativity is characterized by original and breakthrough ideas, involving the development of new ideas that differ significantly from existing processes and practices, and it helps in identifying problems and discovering new approaches during uncertain situations and the early stages of problem solving (Madjar et al., 2011; Ren & Song, 2024). Incremental creativity, in contrast, focuses on making small but novel improvements to existing processes and practices and plays a dominant role in stable environments and during the implementation of solutions and processes (Gilson & Madjar, 2011; St-Louis & Vallerand, 2015). Employees possessing both radical and incremental creativity can effectively respond to changes in the internal and external environments of the organization and enhance their career competitiveness (Gilson & Madjar, 2011; Ren & Song, 2024). Therefore, improving employee radical and incremental creativity is of great significance for employee career development and organizational performance improvement (Qi et al., 2025). In this study, we seek to focus on the relationship between employee self-oriented perfectionism and radical and incremental creativity, and examine under what conditions these associations will be enhanced or diminished.

Among the numerous factors influencing employee creativity, personality and individual traits are often regarded as fundamental antecedents of diverse forms of employee creativity (Shalley et al., 2004; Malik et al., 2019), as the generation of creativity requires sufficient personal resources and internal drive. Self-oriented perfectionism has recently received considerable attention from organizational psychologists, referring to “a tendency to set high standards and pursue perfection for oneself” (Hewitt & Flett, 1991). Along with increasing competition in social and economic environments, more organizations encourage their employees to strive for excellence and higher performance (Bakker & Schaufeli, 2008). Consequently, an increasing number of employees display an increasingly pronounced tendency toward self-oriented perfectionism (Ocampo et al., 2020; Wu et al., 2026), and attention has been given to the impact of self-oriented perfectionism on work outcomes. For example, employee self-oriented perfectionism can influence work engagement (Stoeber & Damian, 2016), burnout (Childs & Stoeber, 2010), taking charge behavior (Wei & Zhang, 2023), well-being (Mohr et al., 2022), career development (Otto et al., 2026), and so on. However, surprisingly, the role of self-oriented perfectionism, a prevalent and positive personality trait in organizations, in shaping individual creativity has been largely overlooked by scholars. This limitation calls for urgent attention, as research has indicated that creativity generation requires “strenuous mental energy” (Shalley et al., 2004, p. 39), enabling individuals to possess sufficient personal resources to engage in high-risk and challenging creative tasks. This suggests that individual traits characterized by energy and motivation toward creative tasks are likely to be important driving factors in predicting creativity (Amabile et al., 1996; Shalley et al., 2004). Following this logic, this paper attempts to examine the relationship between employee self-oriented perfectionism and creativity, given that it is characterized by individuals striving toward preset high goals and persistently and proactively acquiring personal resources (Wigert et al., 2012). Therefore, although perfectionism has rarely been studied in the creativity literature, in this paper, we explore the close relationship between employee self-oriented perfectionism and radical and incremental creativity, as well as its underlying mediating mechanisms and potential boundary conditions.

The model of proactive motivation serves as an appropriate theoretical framework for understanding the relationship between self-oriented perfectionism and radical and incremental creativity. This model centers on the antecedents and consequences of proactive work behavior, proposing that personal characteristics, such as personalities and personal traits, can activate employees’ proactive motivational states, thereby stimulating proactive work behaviors and ultimately fostering favorable work outcomes (Hong et al., 2016; Parker et al., 2010). Building upon this perspective and integrating insights from the job crafting literature, we posit that self-oriented perfectionism is positively related to job crafting, and that job crafting, in turn, is positively associated with both radical and incremental creativity. Prior research indicates that employees with high levels of self-oriented perfectionism tend to be more goal-oriented (Powers et al., 2012), possess a stronger sense of control and self-efficacy (Hart et al., 1998; Trumpeter et al., 2006), and exhibit elevated intrinsic achievement motivation to attain superior performance (Stoeber et al., 2009). These positive attributes enable them to sustain a proactive motivational state at work, thereby generating the willingness, energy, and vigor required to engage in proactive behaviors, specifically, job crafting (Fletcher & Speirs Neumeister, 2012). Job crafting is defined as “the physical and cognitive changes individuals make in the task or relational boundaries of their work” (Wrzesniewski & Dutton, 2001, p. 179). As a kind of proactive work behavior, job crafting reflects self-initiated alterations that better align one’s work with personal needs, goals, values, or interests (Berg et al., 2010). Through job crafting, employees can actively increase job resources that are conducive to creativity (e.g., learning opportunities, autonomy, and feedback seeking) while reducing job demands that are detrimental (e.g., time pressure and negative affect) (Bakker et al., 2012). Accordingly, we propose that job crafting plays a key mediating role in the positive relationship between employees’ self-oriented perfectionism and both radical and incremental creativity.

Furthermore, the model of proactive motivation posits that the activation of employees’ proactive motivational states and behaviors is shaped not only by individual characteristics but also by the interactive effects of contextual factors, such as task characteristics and the work environment (Parker et al., 2010). Specifically, drawing on trait activation theory, we propose that work stress, as a salient perceived work-related contextual factor, may interact with self-oriented perfectionism, thereby moderating the relationships among self-oriented perfectionism, job crafting, and radical and incremental creativity. Trait activation theory elucidates the dynamic interplay between individual personality traits and contextual cues, suggesting that specific external contextual stimuli can activate intrinsic traits, which subsequently manifest in corresponding behaviors (Tett et al., 2013, 2021). Work stress reflects employees’ perceived threat stemming from factors such as high-performance standards and urgent task completion demands (Tsaur & Tang, 2012). Extant research indicates that stressful environments are closely linked to proactive behaviors, as employees facing workplace pressures tend to adopt proactive coping strategies to eliminate or alleviate these stressors and prevent future discrepancies (Fay & Sonnentag, 2002). For perfectionistic individuals, given their strong self-striving and self-actualization goals (Powers et al., 2012), they typically exhibit high levels of personal initiative in the workplace (Wu et al., 2026). We suggest that when confronted with stressful challenges, perfectionistic individuals may be more likely to pursue self-breakthroughs, and this tendency may correspond to higher levels of proactive coping behaviors (Stoeber & Rennert, 2008). Therefore, we suggest that when the level of work stress is higher, the indirect relationship between self-oriented perfectionism, job crafting, and radical and incremental creativity will be stronger.

**Figure 1:**
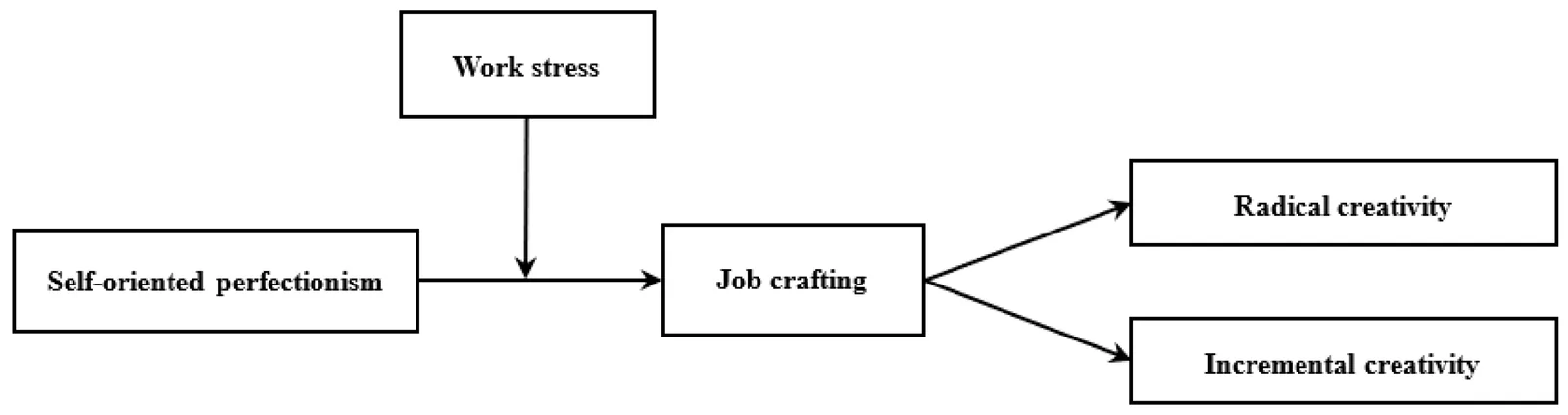
Theoretical framework.

In summary, based on the model of proactive motivation and trait activation theory, we propose a moderated mediation model (as shown in Figure 1) to explore the positive relationship between employees’ self-oriented perfectionism and radical and incremental creativity, as well as the mediating role of job crafting and the boundary conditions of work stress. Our study makes three theoretical contributions. First, we expand the context in which perfectionism can be applied in organizations by exploring the relationship between employees’ self-oriented perfectionism and their radical and incremental creativity, thereby extending research on the consequences of employee perfectionism. Second, based on the model of proactive motivation, we examine the positive relationship between self-oriented perfectionism and employees’ proactive work behavior, and by integrating the job crafting literature, we enrich the motivational mechanism through which perfectionism yields positive outcomes. Finally, we expand the application of trait activation theory by exploring the potential interaction effect between work stress and perfectionism, providing new empirical evidence for exploring the activating contexts of the positive role of self-oriented perfectionism and for testing the positive influence of work stress on employees’ proactive behavior.

## 2. Theory and Hypotheses

### 2.1. The Mediating Role of Job Crafting

The model of proactive motivation provides an important foundation for exploring the process through which employees proactively seek positive work outcomes in organizations. This theory posits that employee proactive behavior is a goal-driven motivational process in which positive personal characteristics (e.g., proactive personality and traits) can facilitate proactive work behaviors for goal generation and goal striving by activating positive motivational states, ultimately producing positive work outcomes (Parker et al., 2010). Therefore, this theory has been widely adopted in organizational scholarship to explain the proactive motivational and behavioral mechanisms through which personal characteristics stimulate positive work outcomes (Parker et al., 2010). As a self-initiated proactive work behavior, job crafting shifts the focus of work design from organizational designers to employees themselves, who can autonomously change their work (Wrzesniewski & Dutton, 2001). It emphasizes that employees can adjust their job content and boundaries through proactive and meaningful behaviors, thereby changing their perception of work meaningfulness or work identity and better coping with the imbalance between job demands and job resources, enhancing their competence in dealing with job tasks (Tims et al., 2013). We argue that because perfectionistic employees set high work standards and goals for themselves (Ocampo et al., 2020), they are prompted to change work processes, working methods, and thinking patterns in their jobs. Thus, we believe that proactive job crafting mediates the relationship between self-oriented perfectionism and radical and incremental creativity.

On the one hand, employees with self-oriented perfectionism are usually confirmed to have higher levels of goal orientation (Stoeber & Otto, 2006), which makes them have “reason to” adjust work tasks, processes, and cognitive patterns, that is, to engage in job crafting. With goal orientation, they are more likely to form stronger achievement motivation and job autonomy (Zhou, 1998), which helps them seek job resources and adjust their work behaviors proactively through seeking feedback and other proactive work behaviors to achieve their goals quickly (I. D. Zhang et al., 2021). Additionally, a higher level of goal orientation can help individuals better implement work plans. By clearly defining when, how, and what plans should be implemented, individuals can handle work setbacks and obstacles more smoothly. They will adjust their working methods by job crafting according to their work plans to achieve the ultimate perfection of work goals. High goal orientation can also bring about positive goal-related mindstreams for individuals, resulting in stronger intrinsic motivation and more positive affective resources (Vansteenkiste et al., 2010), which can promote proactive adjustments to work styles and increased productivity.

On the other hand, employees with self-oriented perfectionism usually have more positive cognitions and attitudes toward their jobs, which makes them “energize to” engage in job crafting. First, perfectionists are usually good at reflection, which helps them better understand their personal needs and goals, thereby enhancing their efforts related to work tasks (Messmann, 2023). By reflecting on problems and difficulties encountered at work, employees who are perfectionists will take proactive measures to improve in relevant areas (i.e., learning new skills and expanding their social interaction; Q. Wang et al., 2023). Second, individuals who pursue perfection are extremely afraid of failure (Lin et al., 2023). To avoid failure, they can actively check work details to avoid errors in their work, which enables them to increase their level of problem-solving creativity in response to errors at work. Finally, they hold high standards and stringent requirements for their own work performance and outcomes (Stoeber & Childs, 2010), which necessitates that they continuously modify their work processes and approaches, as well as adjust their work tasks and schedules, in order to attain optimal performance. In summary, we propose the following:

**Hypothesis** **1.** *Self-oriented perfectionism has a positive relationship with job crafting.*

The model of proactive motivation suggests that individuals can effectively facilitate the achievement of positive work outcomes through the goal-striving process of engaging in proactive work behaviors (Parker et al., 2010). We argue that job crafting can increase work resources (Bakker et al., 2012), enable employees to obtain positive emotional experiences (Van den Heuvel et al., 2015) and improve employees’ work engagement (Tims et al., 2012). Previous studies have shown that employees can stimulate their positive affective states through job crafting (Shi et al., 2022). At this time, employees are more likely to show flexible, creative and unusual thinking (Lee & Sun, 2022), which is more conducive to employees’ radical creativity. In the meantime, the more individual resources employees obtain through job crafting, the stronger their willingness to work (Bakker & Demerouti, 2017), which means that they will probably show a higher enthusiasm for work. Thus, employees are willing to invest their resources, such as knowledge and skills, in creative activities (W. Zhang et al., 2017). This sustained investment of resources is particularly conducive to their incremental creativity, as it supports continuous refinements that are grounded in existing knowledge.

In addition, job crafting can reduce job demands (Bakker et al., 2012), relieve employees’ negative affective states (Demerouti, 2014) and reduce job burnout (Böhnlein & Baum, 2022; Cheng & Yi, 2018). It helps to alleviate the hostile and tense atmosphere in the workplace (Demerouti, 2026; Deng et al., 2020), enabling employees to eliminate interpersonal concerns and dare to make mistakes, realizing the improvement of their radical creativity. Meanwhile, by continuously optimizing their daily workflows and methodologies, employees can harvest a steady stream of novel inspirations and ideas during their routine work (Uen et al., 2021). Furthermore, leveraging enriched structural job resources enables employees to constantly reshape their perspectives and understanding of their work, which in turn facilitates incremental creativity aimed at the ongoing refinement of their tasks (Ren & Song, 2024). In summary, we propose the following:

**Hypothesis** **2.** *Job crafting has a positive relationship with (a) radical creativity and (b) incremental creativity.*

**Hypothesis** **3.** *Job crafting mediates the positive indirect relationship between self-oriented perfectionism and (a) radical creativity and (b) incremental creativity.*

### 2.2. The Moderating Role of Work Stress

The model of proactive motivation suggests that contextual factors can interact with personal characteristics to jointly influence the generation process of individual proactive motivation and behavior (Parker et al., 2010), which prompts us to pay attention to the interactive effects of contextual factors and perfectionism. Considering that trait activation theory posits that personality traits are latent dispositional tendencies that translate into explicit work or daily behaviors only when individuals encounter trait-relevant “situational cues” (Tett & Burnett, 2003), and given that prior research has also pointed out that perfectionism is an individual’s inner potential, with specific contextual conditions exerting stronger driving or inhibiting effects on perfectionistic individuals’ cognition, affect, and behavioral performance (Ocampo et al., 2020), we need to further explore the contextual boundary conditions in the process through which self-oriented perfectionism influences radical and incremental creativity. As an important source of promoting individual proactivity, we argue that work stress serves as a key contextual factor that activates the motivation of self-oriented perfectionistic employees to pursue perfection and high standards. In order to close the gap between actual work performance and their high standards, self-oriented perfectionistic employees under work stress are more motivated to engage in job crafting, which will have a stronger relationship with their radical and incremental creativity.

First, work stress is widely regarded in organizational scholarship as an important antecedent that drives individual proactivity (Fay & Sonnentag, 2002). Work stress typically signals that an individual’s current operating processes, work environment, or efficiency are below optimal levels, indicating that there are problems to be addressed (Schwarzer & Reuter, 2023). Driven by anticipation of the future and the pursuit of efficient work, individuals transform stress into forward-looking thinking, and subsequently take proactive actions to improve the environment, remove obstacles, or enhance efficiency, such as engaging in job crafting (Fay & Sonnentag, 2002; Sonnentag & Spychala, 2012). Perfectionistic individuals, who hold strong beliefs and motivation to achieve high goals and work standards, are more likely to adopt proactive coping strategies when facing work stress, so as to help themselves quickly attain their expected work goals (Ocampo et al., 2020). Moreover, work stress may induce individuals to anticipate the possibility of future failure (Biron et al., 2010). In order to eliminate such potential deviation and prevent future setbacks, individuals are activated to take proactive and long-term-oriented actions. Perfectionistic employees, who are afraid of failure (Lin et al., 2023), are therefore more likely to be activated to strive for future positive outcomes and to avoid failure to the greatest extent through work effort. Consequently, they mobilize their job resources when facing stress and take proactive measures to avoid failure (Neumeister, 2004), such as proactively learning new skills and developing new working relationships, to ensure that they can survive in a high-pressure environment and make no mistakes.

Furthermore, research indicates that perfectionistic individuals with positive traits are more likely to adopt proactive coping strategies when facing negative situations such as stress or failure (Larijani & Besharat, 2010). When confronted with negative events or stress, the psychological mechanisms and coping strategies of perfectionists are activated. For employees with high levels of self-oriented perfectionism, stress or challenges are not impediments; rather, work stress is often perceived as an expectation and recognition of their abilities (Prem et al., 2017), which provides them with opportunities for learning and growth. Their trait of pursuing high standards and goals also drives them to adopt job crafting as a coping strategy to change the status quo and improve outcomes, which effectively mitigates the negative impact of work stress on themselves (Stoeber & Janssen, 2011; Stumpf et al., 1987). Moreover, when employees with self-oriented perfectionism believe that the time and energy they invest in dealing with work stress can be rewarded in a meaningful way, they increase their work commitment (Macey & Schneider, 2008) and become more proactive in dealing with challenging situations, such as being more proactive in seeking out resources and proactively adapting their work style. Thus, work stress is an important contextual factor that activates perfectionistic employees’ adoption of job crafting as a coping strategy. Therefore, we propose the following hypothesis:

**Hypothesis** **4.** *Work stress moderates the relationship between self-oriented perfectionism and job crafting. Specifically, when the level of stress is higher, the positive relationship between self-oriented perfectionism and job crafting is stronger.*

### 2.3. An Integrated Model

In sum, job crafting is a crucial mediator linking employees’ self-oriented perfectionism and radical and incremental creativity. Specifically, when employees have a high level of self-oriented perfectionism, they are more likely to proactively engage in job crafting, which in turn promotes their radical and incremental creativity. As a key contextual factor, work stress can motivate employees with self-oriented perfectionism to generate higher levels of job resources, thereby motivating them to engage in higher levels of job crafting and bringing about higher levels of radical and incremental creativity.

**Hypothesis** **5.** *Work stress moderates the positive indirect relationships between self-oriented perfectionism and (a) radical creativity and (b) incremental creativity. Specifically, the mediated relationships are stronger at higher levels of work stress.*

## 3. Method

### 3.1. Participants

In this study, we employed a three-wave, time-lagged design to collect data from frontline teachers in primary and secondary schools in China. We first contacted school administrators and invited their schools to participate in the study. With their assistance, full-time frontline teachers were invited to complete the survey. A total of 933 frontline teachers were invited to participate in the study. Before completing the questionnaires, participants were informed of the purpose of the research and assured that participation was voluntary and that all responses would remain anonymous and confidential. To match responses across the three waves while preserving anonymity, each participant was assigned a unique identification code.

We finally obtained 778 matched responses with a response rate of 83.39%. Specifically, 25.6% (199) of the samples were male, and 74.4% (579) were female. Regarding age, 8.1% were 25 years old or younger, 33.5% were 25–35, 22.2% were 35–45, and 36.1% were 46 or older. In terms of education level, 2.3% had college degrees or below, 80.1% had bachelor’s degrees, 16.8% had master’s degrees, and 0.8% had doctoral degrees. Regarding tenure, 33.3% of the participants had worked for 3 years or less, 15.0% for four to seven years, and 51.7% had worked for more than 7 years. In terms of annual salary, 10.4% of the participants were under RMB 50,000, 28.1% earned RMB 50,000–100,000, 50.6% earned RMB 100,000–200,000, and 10.8% earned above RMB 200,000.

### 3.2. Measures

All English scales were translated into Chinese following Brislin’s (1980) back-translation method. We used a five-point Likert scale for all measures ranging from 1 (strongly disagree) to 5 (strongly agree) unless otherwise specified. Detailed items were provided in Appendix A. Reported Cronbach’s alphas were calculated using the present sample.

#### 3.2.1. Self-Oriented Perfectionism

We utilized the self-oriented perfectionism subscale with five items taken from the multidimensional perfectionism scale developed by Hewitt and Flett (1991), referring to previous studies (Zeijen et al., 2018). The original seven-point response format was adapted to a five-point Likert scale to maintain consistency across the questionnaire, as five- and seven-point formats generally show comparable response characteristics (Dawes, 2008; Robinson, 2018). A sample item of the scale is “One of my goals is to be perfect in everything I do”. The Cronbach’s alpha is 0.81.

#### 3.2.2. Job Crafting

We measured employees’ job crafting using Tims et al.’s (2012) 21-item scale which comprised four dimensions: increasing structural job resources (5 items), increasing social job resources (5 items), increasing challenging job demands (5 items), and decreasing hindering job demands (6 items). Participants rated each item on a five-point frequency scale ranging from 1 (never) to 5 (often). A sample item of the scale is “I try to learn new things at work”. The Cronbach’s alpha is 0.95.

#### 3.2.3. Radical Creativity and Incremental Creativity

Both types of creativity were assessed by the scale developed by Madjar et al. (2011). The scale comprised two dimensions, radical creativity and incremental creativity, with three items for each dimension. The radical and incremental creativity items were adapted to the education and teaching context while preserving their original conceptual meaning. Specifically, the original third-person wording was adjusted to the first person to reflect respondents’ own teaching-related behaviors, without changing the substantive content of the items. Minor wording adjustments were also made to improve grammatical accuracy and contextual consistency. All items were employee self-rated and based on a seven-point Likert scale ranging from 1 (strongly disagree) to 7 (strongly agree). A sample item of radical creativity is “I am a good source of highly creative ideas”, with a Cronbach’s alpha of 0.94. A sample item of incremental creativity is “I use previously existing ideas or work in an appropriate new way”, with a Cronbach’s alpha of 0.94.

#### 3.2.4. Work Stress

We used H. L. Wang and Zhang’s (2016) 3-item scale as adapted from Motowidlo et al. (1986). A sample item is “I feel a great deal of stress because of my job”. The Cronbach’s alpha is 0.93.

#### 3.2.5. Control Variables

The personal characteristics of individuals may affect their behaviors. Based on previous research on workplace perfectionism, job crafting and creativity, some demographic variables (including gender, age, education level, tenure and annual salary) were taken as control variables (Tims et al., 2012; Madjar et al., 2011).

### 3.3. Data Collection Procedure

All study variables were measured using self-reported data. The same pool of participants was invited to participate at each of the three survey waves, with a two-week interval between consecutive waves. At Time 1, participants reported their self-oriented perfectionism, perceived work stress, and demographic information. Two weeks later, at Time 2, they reported their job crafting behaviors. After another two weeks, at Time 3, they reported their radical and incremental creativity. Within each wave, all participants completed the measures in the same fixed order. Each survey took approximately 5–10 min to complete. Participation across waves was not necessarily sequential, as some individuals responded to certain waves but not others. Therefore, only participants who completed all three waves and whose responses could be successfully matched were retained in the final sample. Responses showing clear signs of low response quality (e.g., selecting the same response option across all items) were also excluded.

### 3.4. Statistical Procedures

All analyses were conducted using SPSS 26.0 and Mplus 8.3. SPSS 26.0 was used to obtain descriptive statistics, Cronbach’s alphas for internal consistency reliability, and Pearson correlations to assess bivariate associations among the study variables. Harman’s single-factor test was also conducted in SPSS 26.0 to assess potential common method bias. The reliability and validity of the study constructs were further evaluated using Mplus 8.3. Confirmatory factor analyses (CFAs) were conducted to assess construct distinctiveness by comparing the hypothesized five-factor model with alternative four-, three-, two-, and single-factor models. Composite reliability (CR) and average variance extracted (AVE) were used to assess construct reliability and convergent validity, respectively. Discriminant validity was evaluated using the Fornell–Larcker criterion and the heterotrait–monotrait ratio (HTMT).

For hypothesis testing, hierarchical regression analyses were conducted in SPSS 26.0 to examine the proposed relationships among self-oriented perfectionism, job crafting, and radical and incremental creativity. The indirect relationship between self-oriented perfectionism, job crafting, and radical and incremental creativity was tested using bootstrapping with 5000 resamples. The moderating effect of work stress on the relationship between self-oriented perfectionism and job crafting was examined using interaction terms and simple slope analyses with PROCESS for SPSS (Hayes, 2017). Finally, Mplus 8.3 was used to estimate the conditional indirect effects, the index of moderated mediation, and the difference between the indirect effects on radical and incremental creativity.

## 4. Results

### 4.1. Preliminary Analysis

To examine potential attrition bias, we tested for group differences in demographic variables. Retained and non-retained participants did not differ in age, where χ^2^(3) = 3.697, *p* = 0.296, and Cramér’s V = 0.063; education, where χ^2^(1) = 3.355, *p* = 0.067, and Cramér’s V = 0.060; tenure, where χ^2^(2) = 4.887, *p* = 0.087, and Cramér’s V = 0.072; or annual salary, where χ^2^(3) = 7.073, *p* = 0.070, and Cramér’s V = 0.087. To avoid sparse expected cell counts, education was recoded into two categories: bachelor’s degree or below and master’s degree or above. A significant difference was found only for gender, where χ^2^(1) = 17.044, *p* < 0.001, and Cramér’s V = 0.135. Although a significant gender difference was observed, the magnitude of this difference was relatively limited (Cramér’s V = 0.135), with the value close to the conventional benchmark of 0.10 for a small effect. Together with the nonsignificant differences in age, education, tenure, and annual salary, these findings suggest limited demographic selectivity in attrition.

As shown in Table 1, CRs (construct reliabilities) of all variables were above the reference value of 0.70, indicating that all the scales used in this paper have good reliability. Simultaneously, the AVE (average variance extracted) values of all measurements reached the threshold of 0.50, which meant that the convergent validity of all scales was also acceptable. Moreover, the square root of AVE was larger than the correlation coefficient between variables, illustrating that the discriminant validity also reached the standards. Because of the relatively high correlation between radical and incremental creativity, we further assessed their discriminant validity using the heterotrait–monotrait ratio of correlations (HTMT). As shown in Table 2, all HTMT values were below the recommended threshold of 0.90 (Henseler et al., 2015). The highest value was 0.86 between radical and incremental creativity, providing additional evidence of discriminant validity.

We also conducted confirmatory factor analysis (CFA) to verify the discriminant validity among the model’s main variables as shown in Table 3. The results (*χ*^2^/*df* = 3.46; CFI = 0.92; TLI = 0.91; RMSEA = 0.06; SRMR = 0.06) showed that our theoretical model fits the data better than the alternative models, demonstrating that the main variables have acceptable discriminant validity. To further examine the distinction between radical and incremental creativity, we compared a two-factor model with an alternative one-factor model. The two-factor model showed a good fit to the data (*χ*^2^ = 19.855, *df* = 8, CFI = 0.996, TLI = 0.992, RMSEA = 0.044, SRMR = 0.011) and fit significantly better than the one-factor model (*χ*^2^ = 220.652, *df* = 9, CFI = 0.925, TLI = 0.875, RMSEA = 0.174, SRMR = 0.043; Δ*χ*^2^ = 200.767, Δ*df* = 1, *p* < 0.001). All items loaded significantly on their intended factors. Given that all study variables were assessed using self-reported questionnaires, we conducted Harman’s single-factor test to assess the potential influence of common method bias. The first unrotated factor accounted for 35.87% of the total variance, which was below the commonly used 40% threshold. The results indicate that common method bias does not pose a significant concern in this study.

### 4.2. Testing Mediating Effects

As shown in Table 4, Model 2 indicates that self-oriented perfectionism was positively associated with job crafting (*b* = 0.24., *p* < 0.001). Models 6 and 9 further show that self-oriented perfectionism was significantly associated with radical creativity (*b* = 0.47, *p* < 0.001) and incremental creativity (*b* = 0.38, *p* < 0.001). Meanwhile, after job crafting was included in Models 7 and 10, the associations of self-oriented perfectionism with radical creativity (*b* = 0.28, *p* < 0.001) and incremental creativity (*b* = 0.21, *p* < 0.001) remained significant. Job crafting was also significantly associated with radical creativity (*b* = 0.79, *p* < 0.001) and incremental creativity (*b* = 0.71, *p* < 0.001). Moreover, the direct associations between self-oriented perfectionism and both forms of creativity remained significant after job crafting was included in the models, indicating that job crafting partially mediated these relationships. To further confirm the mediating effect, we conducted a bootstrap analysis. Specifically, 5000 bootstrap samples were randomly selected by repeated random sampling, and the 95% confidence interval was obtained through the estimated value of the mediation effect. The indirect relationship between self-oriented perfectionism and radical creativity and incremental creativity via job crafting is 0.19 (LLCI = 0.13, ULCI = 0.26) and 0.16 (LLCI = 0.11, ULCI = 0.22), respectively, and the 95% confidence intervals both excluded 0 and reached a significant level. Thus, Hypotheses 1, 2 and 3 are supported.

### 4.3. Testing Moderating Effects

**Figure 2:**
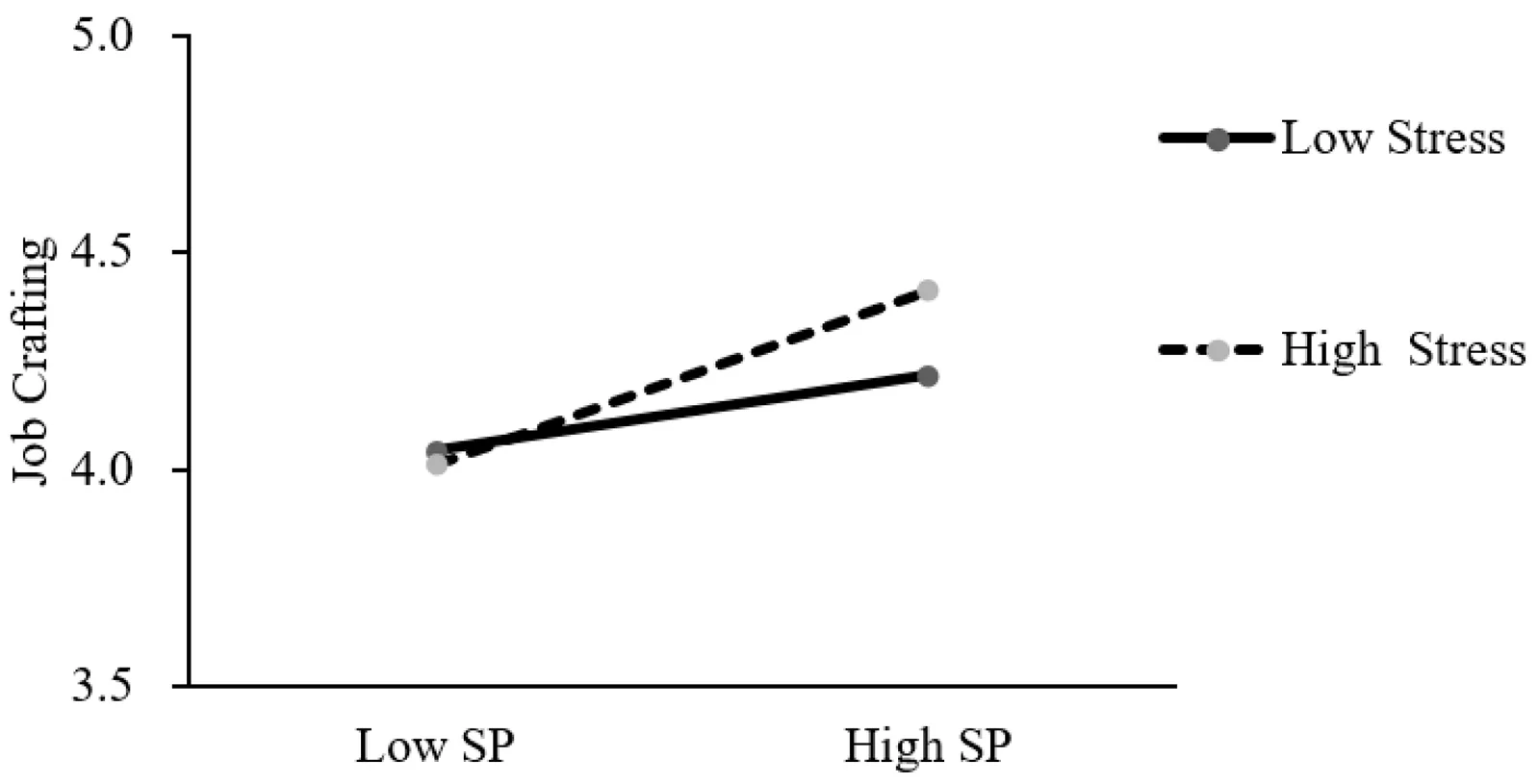
The moderating role of work stress. Note. SP = self-oriented perfectionism. Stress = work stress.

We also tested the interaction effect as manifested in Table 4. Model 3 shows that work stress is significantly related to job crafting (*b* = 0.04, *p* < 0.05). Model 4 shows that the interaction term (self-oriented perfectionism × work stress) is positively associated with job crafting (*b* = 0.06, *p* < 0.001), suggesting that stress plays an important moderating role in the relationship between self-oriented perfectionism and job crafting. To further interpret the moderating effect, we analyzed the different relationships between self-oriented perfectionism and job crafting under different levels of work stress. As shown in Table 5, when work stress is at a low level (MEAN − 1 SD), the relationship between self-oriented perfectionism and job crafting is significantly positive with a coefficient of 0.14 (*p* < 0.001). The relationship is still significantly positive at the mean level (*p* < 0.001), and the coefficient increases to 0.23. In addition, when work stress is at a high level (MEAN + 1 SD), it is still significant (*p* < 0.001), and the coefficient reaches 0.32. As shown in Figure 2, the association between self-oriented perfectionism and job crafting is gradually strengthened with the increasing level of work stress. Thus, Hypothesis 4 is supported.

Then, we tested the index of moderated mediation (Hayes, 2015) to examine the complete path from self-oriented perfectionism to radical creativity and incremental creativity via job crafting. Table 6 shows that work stress is grouped into low (MEAN − 1 SD) and high (MEAN + 1SD) levels. When work stress is low, both mediating paths are significant, yet relatively weak (radical creativity: effect = 0.08, LLCI = 0.03, ULCI = 0.14; incremental creativity: effect = 0.07 LLCI = 0.02, ULCI = 0.12). When work stress is high, the 95% CIs of the mediating effects, from self-oriented perfectionism to radical creativity (LLCI = 0.13, ULCI = 0.24) and incremental creativity (LLCI = 0.11, ULCI = 0.20) via job crafting, do not include zero. The coefficients for these two paths are 0.18 and 0.15, respectively. The differences between the high and the low levels are 0.10 (LLCI = 0.04, ULCI = 0.16) and 0.09 (LLCI = 0.03, ULCI = 0.14), respectively. The moderated mediating effect indices are 0.05 (radical creativity) and 0.04 (incremental creativity), and the 95% CI (LLCI = 0.02, ULCI = 0.08; LLCI = 0.02, ULCI = 0.07) does not include 0. Therefore, both of the indices are significant, which means that moderated mediated effects exist; thus, Hypothesis 5 is also supported.

### 4.4. Supplementary Analysis

We also compared the mediating associations of the two paths from self-oriented perfectionism to radical creativity and incremental creativity. The results showed that, regardless of the level of work stress, self-oriented perfectionism had a greater association with radical creativity than with incremental creativity. As work stress levels increased, this difference gradually amplified. Specifically, when work stress was at a low level, the indirect relationship between self-oriented perfectionism and radical creativity was stronger than incremental creativity, and the difference was 0.01 and marginally significant (*p* < 0.1, 95% CI [0.00, 0.03]). When the work stress was at the mean level, the difference was 0.02 and significant (*p* < 0.01, 95% CI [0.01, 0.04]). We will further explain this phenomenon in the discussion. When the work stress reached a high level, the difference increased to 0.03 and significant (*p* < 0.01, 95% CI [0.01, 0.05]).

## 5. Discussion

This study aimed to explore the mediating mechanism and boundary condition in the relationship between self-oriented perfectionism and radical and incremental creativity of employees, and the proposed research model was tested through a multi-time survey. All of the research hypotheses were fully supported by the empirical results. First, we found that employees’ self-oriented perfectionism is positively associated with both radical and incremental creativity. This finding is consistent with prior research, which suggests that employees with high levels of perfectionism tend to strive for achievement and progress, and this tendency may be linked to higher creative performance (S. L. Kim et al., 2017). Second, we found that job crafting mediates the positive relationship between self-oriented perfectionism and radical and incremental creativity. This result further corroborates the role of stable personality traits in predicting job crafting (Bipp & Demerouti, 2015; Laguía et al., 2024), aligns with existing views on how job crafting triggers individual creativity (H. Li et al., 2020), and thus enriches the literature on both the antecedents and consequences of job crafting. Finally, the results confirmed the moderating role of work stress in the process through which self-oriented perfectionism relates to job crafting and, subsequently, to radical and incremental creativity. Consistent with previous studies, this finding not only reaffirms the predictive role of work stress in facilitating individual work proactivity (Fay & Sonnentag, 2002; Searle, 2008), but also provides additional empirical evidence for the contextual role of stressful situations and events in activating perfectionistic traits among employees (Petrou et al., 2015; Pletneva, 2024). In the following section, we will discuss in depth the theoretical contributions, practical contributions, limitations, and future research directions of this study.

### 5.1. Theoretical Implications

Our study makes theoretical contributions in three ways. First, we expanded the understanding of the personality antecedents of employee creativity in organizational contexts. Some organizational psychologists have confirmed that personality is an antecedent affecting employee creativity (e.g., narcissism, Mao et al., 2021; Big Five personality traits, Yao & Li, 2021; proactive personality, T. Y. Kim et al., 2009). However, surprisingly, perfectionism—a common personality trait in the workplace—has been largely overlooked, even though it has been recognized as increasingly prevalent in organizations over the past two decades (Ocampo et al., 2020). Although organizational scholars have recently paid increasing attention to the effect of leader perfectionism on employee creativity, this stream of research has neglected the role of individual agency in the activation process of employee creativity. A few studies have focused on the direct effect of employee self-oriented perfectionism on creativity (e.g., Hu et al., 2026; Ocampo et al., 2020; Xu et al., 2022), yet they have not thoroughly examined its underlying mechanisms and boundary conditions. By examining the relationship between self-oriented perfectionism and radical and incremental creativity, our study aligns with previous research suggesting that perfectionism, as a typical workplace personality trait, can lead to positive performance outcomes through the setting of high standards and ambitious goals (Wu et al., 2026). Therefore, our study focuses on employees themselves and further enriches the research on the impact of perfectionism on different types of creativity.

Interestingly, by comparing the mediating associations of the two paths from self-oriented perfectionism to radical creativity and incremental creativity, we found that the indirect relationship between self-oriented perfectionism and radical creativity was stronger than that for incremental creativity, and this difference widened as work stress increased. This indicates that under stressful work situations, the association between self-oriented perfectionism and radical creativity via job crafting tends to be stronger than that for incremental creativity. We argue that the personality traits and behavioral preferences of perfectionistic individuals may be consistent with these differential associations. On the one hand, from the exploration-exploitation perspective of creativity generation, radical and incremental creativity typically represent exploratory and exploitative processes (Gilson & Madjar, 2011; C. R. Li et al., 2019), respectively. Exploitative processes tend to yield only incremental improvements by utilizing existing resources, which often fall short of the high standards set by self-oriented perfectionists (De Visser & Faems, 2015). In contrast, in pursuit of substantial breakthroughs, perfectionistic individuals may be dissatisfied with standard procedures and may also actively seek additional resources and exploratory processes (Ocampo et al., 2020). On the other hand, from the perspective of cognitive characteristics, perfectionistic individuals possess high cognitive flexibility (Hayatbini et al., 2021). When facing difficult problems at work, in order to achieve high standards, they may examine work tasks from a more holistic and disruptive perspective, which is conducive to breaking existing thought patterns and may contribute to breakthrough innovative ideas (Goulet-Pelletier et al., 2022).

Second, our research reveals the potential positive nature of employee self-oriented perfectionism, further unveiling the mechanisms through which perfectionism links with positive effects. To date, the majority of organizational scholars have considered the impact of employee self-oriented perfectionism on employees as a double-edged sword (Ocampo et al., 2020). For example, self-oriented perfectionism may lead to work addiction (Morkevičiūtė & Endriulaitienė, 2022) and workplace cheating (K. Zhang & Li, 2020), but it can also reduce employee burnout (Childs & Stoeber, 2010) and increase organizational citizenship behavior (Y. C. Wang et al., 2021). These inconsistent findings have confounded the understanding of self-oriented perfectionism in organizational scholarship. Although research has recognized its motivational role in positively driving employees’ proactive work behaviors, few studies have empirically examined this proposition. Based on the model of proactive motivation and trait activation theory, and from a personal initiative perspective, we examined the interactive effects of self-oriented perfectionism and work stress on creativity. This is consistent with prior research and responds to the emphasis by creativity scholars on the benefits of the interplay between individual traits and contextual factors in promoting employee creativity (Shalley & Gilson, 2004). In summary, our study reveals the potential positive effects of self-oriented perfectionism from a motivational perspective, and answers the call by Wu et al. (2026) for future research to pay greater attention to the role of perfectionism traits in influencing individual work behaviors and performance, thereby enriching the literature on perfectionism, creativity, and job crafting.

Finally, we point out the key role of work stress in shaping the creativity of perfectionistic employees and explore its crucial role in activating their proactive behavior. We respond to the call of Ocampo et al. (2020) and explain the positive role of work stress for perfectionistic employees. Numerous existing studies have primarily focused on the direct impact of perfectionism on state stress (e.g., Dunkley et al., 2003). However, with the changing business environment, it is necessary to consider the boundary condition of organizational work stress on workplace perfectionism. Although scholars have already pointed out the enhancing relationship between stress context and individual coping proactivity (Fay & Sonnentag, 2002; J. Park et al., 2012; Stoeber & Rennert, 2008) and work motivation (Kongcharoen et al., 2020), few studies have focused on its positive interactive effects with individual characteristics. Moreover, psychological research on perfectionism has also pointed out the potential role of stressful events in enhancing individual performance striving, yet organizational scholarship has seldom integrated perfectionism with work stress to understand the activating contexts under which perfectionism exerts positive effects on work outcomes. By integrating employee self-oriented perfectionism with work stress contexts, our research reveals the crucial contextual role of work stress in the process of perfectionistic employees engaging in proactive behavior, which provides a new perspective for trait activation theory used in perfectionism research. It is worth noting that, although the findings of this study are consistent with those of prior research in suggesting that work stress may, to a certain extent, be associated with individual proactivity and job crafting, this conclusion applies only to the potential positive effects of general work stress as conceptualized and measured in this study. Given that work stress manifests in diverse forms (Barling et al., 2004) and that individuals’ cognitive appraisals of stress as either positive or negative (e.g., challenge appraisal versus hindrance appraisal) may lead to divergent outcomes (Podsakoff et al., 2007), some studies have also pointed out that work stress may be associated with individual proactivity only under certain contextual conditions. For instance, contextual factors such as organizational HR practices, colleague support, and family support, which are forms of social support, can help employees cope with work stress and thereby encourage them to proactively address and resolve stressful events (Viswesvaran et al., 1999). Therefore, the positive moderating effect of work stress identified in this study is specific to the general work stress construct employed herein and should not be generalized to all types or dimensions of stress.

### 5.2. Practical Implications

First, managers should be aware that perfectionism does not always have negative impacts on organizational effectiveness. In some contexts, self-oriented perfectionism may be associated with higher levels of radical and incremental creativity. Meanwhile, both organizations and employees should recognize that work stress is not necessarily detrimental and, under certain conditions, may stimulate employees to respond more proactively to work demands and engage in creative activities. In particular, when employees experience higher work stress, managers can provide greater autonomy, resources, and support to help them respond constructively through job crafting. Organizations can also encourage employees to actively adjust their tasks, seek necessary resources, and improve their work methods. Such practices may help employees channel their high performance standards and demanding work experiences into proactive job crafting and sustain both radical and incremental creativity.

### 5.3. Limitations and Future Research Directions

First, we designed a multi-wave survey to reduce the risk of the common method bias (CMB) issue. However, we did not make reasonable causal inferences. Experimental research methods can be further applied in future studies. Meanwhile, because the measures within each wave were administered in a fixed order, potential order effects cannot be completely ruled out. Future research could randomize the order of measures within each wave to further reduce this concern. Additionally, a limitation is that the two-week interval may not fully capture longer-term effects. Future research could examine the proposed relationships over longer time intervals. Second, we used work stress as a moderating variable but defined work stress broadly and did not distinguish between different types of work stress. In fact, work stress can be categorized as being triggered by challenge stressors versus hindrance stressors (Mazzola & Disselhorst, 2019). When individuals appraise work stress as hindrance stress, it may lead to maladaptive work performance, which could weaken the relationship between perfectionism, job crafting, and radical and incremental creativity (LePine, 2022). Future research could further clarify the different types and nature of work stress and explore their distinct interactions with perfectionism. In addition to distinguishing among different types of work stress, future research could further investigate which contextual factors may interact with work stress to jointly produce interaction effects. For instance, studies could explore how positive contextual factors, such as organizational HR practices, coworker support, and family support, can help perfectionist employees effectively cope with stressful work environments (Terry et al., 1993), thereby enhancing the positive relationship between self-oriented perfectionism and job crafting. Third, our study still lacks control over certain additional variables that may influence the research hypotheses. For instance, employee job crafting and creativity may be jointly affected by positive personality traits (e.g., proactive personality, conscientiousness, and openness to experience) as well as contextual factors at both the organizational and team levels (e.g., job autonomy, innovation climate, and supervisory support) (Baas et al., 2013; Bipp & Demerouti, 2015; Oldham & Cummings, 1996; S. Park & Park, 2023). Therefore, beyond the key demographic information controlled for in this study, it remains necessary to account for important individual and contextual factors that may exert an impact on job crafting and both radical and incremental creativity. Fourth, in this study, radical and incremental creativity was assessed through self-reports, although the original scale developed by Madjar et al. (2011) was designed for external ratings. Therefore, although the results of this study suggest that common method bias is not a prominent concern, the use of self-rated creativity measures may still introduce potential common method bias and could inflate the observed associations among self-oriented perfectionism, job crafting, and radical and incremental creativity. Consequently, the effect sizes reported here should be interpreted with caution, as they may overestimate the true relationships. Future research could use supervisor or colleague ratings of creativity to further validate our findings. Finally, the samples of our study were all from mainland China, so the conclusions obtained may not be fully valid in other cultural contexts. Subsequent researchers may consider exploring and expanding this question in different cultural contexts.

## 6. Conclusions

Based on the model of proactive motivation and trait activation theory, this paper explores the relationships between self-oriented perfectionism and both radical and incremental creativity. Through a three-wave questionnaire survey of 778 teachers from China, we found that self-oriented perfectionism has positive relationships with both radical and incremental creativity. Moreover, job crafting mediates these relationships, and work stress, as a boundary condition, moderates the above mediating effects, such that they are stronger at higher levels of work stress. Interestingly, we also found that self-oriented perfectionism has a stronger relationship with radical creativity than with incremental creativity. This finding suggests that perfectionistic individuals, when facing creative demands, may adopt exploratory strategies, which could be associated with a greater degree of creativity and potentially more breakthrough outcomes. In addition, our study enriches the research on the positive role of work stress in relation to creativity among perfectionistic employees, and further supports the notion that work stress is associated with personal initiative and proactive work behaviors. Overall, this paper makes important contributions to the literature on workplace perfectionism, employee proactivity and creativity.

## Figures and Tables

**Table 1:** Descriptive statistics, correlations, reliability and discriminant validity.

Variables	Mean	*SD*	CR	AVE	Discriminant Validity				
					6	7	8	9	10
1 Gender	1.74	0.44							
2 Age	2.86	1.00							
3 Education level	2.16	0.44							
4 Tenure	2.18	0.90							
5 Salary	2.62	0.81							
6 Self-oriented perfectionism	3.72	0.62	0.83	0.51	**0.71**				
7 Job crafting	4.18	0.52	0.86	0.61	0.28	**0.78**			
8 Work stress	3.62	0.92	0.93	0.82	0.17	0.12	**0.91**		
9 Radical creativity	4.89	1.15	0.94	0.84	0.28	0.41	0.06	**0.92**	
10 Incremental creativity	5.25	1.00	0.93	0.82	0.28	0.39	0.02	0.81	**0.91**

Note. *N* = 778. The lower triangle in the matrix is the Pearson correlation coefficient between study variables, and the bold value on the diagonal is the square root of AVE. CR = construct reliability; AVE = average variance extracted. Coding: gender (1 = male, 2 = female), age (1 = 25 years old or below, 2 = 25–35 years old, 3 = 35–45 years old, 4 = 45 years old or above), education level (1 = college degree or below, 2 = bachelor’s degree, 3 = master’s degree, 4 = doctoral degree), tenure (1 = 3 years or below, 2 = 3–7 years, 3 = 7 years above), and annual salary (1 = 50,000 RMB or below, 2 = 50,000–100,000, 3 = 100,001–200,000 RMB, 4 = 200,000 RMB or above).

**Table 2:** Heterotrait–monotrait ratio (HTMT) matrix.

Variables	1	2	3	4
1. Self-oriented perfectionism				
2. Job crafting	0.33			
3. Work stress	0.19	0.13		
4. Radical creativity	0.33	0.43	0.07	
5. Incremental creativity	0.34	0.43	0.04	0.86

Note. *N* = 778.

**Table 3:** Model fit results for confirmatory factor analyses.

Models	χ^2^	*df*	χ^2^/*df*	CFI	TLI	SRMR	RMSEA
5-factor model	1890.77	546.00	3.46 ***	0.92	0.91	0.06	0.06
4-factor model	2199.34	550.00	4.00 ***	0.90	0.90	0.06	0.06
3-factor model	3499.25	553.00	6.33 ***	0.83	0.81	0.08	0.08
2-factor model	9703.73	559.00	17.36 ***	0.46	0.43	0.13	0.15
Single factor model	10,517.73	560.00	18.78 ***	0.41	0.38	0.14	0.15

Note. *N* = 778. CFI = comparative fit index; TLI = Tucker–Lewis index; RMSEA = root mean squared error of approximation; SRMR = standardized root mean-square residual. *** *p* < 0.001. 5-factor model: self-oriented perfectionism, job crafting, work stress, radical creativity, incremental creativity; 4-factor model: self-oriented perfectionism, job crafting, work stress, radical creativity + incremental creativity; 3-factor model: self-oriented perfectionism + work stress, job crafting, radical creativity + incremental creativity; 2-factor model: self-oriented perfectionism + work stress, job crafting + radical creativity + incremental creativity; single-factor model: self-oriented perfectionism + work stress + job crafting + radical creativity + incremental creativity.

**Table 4:** Hierarchical regression analyses results.

Variables	Job Crafting				Radical Creativity			Incremental Creativity		
	M1	M2	M3	M4	M5	M6	M7	M8	M9	M10
Control variables										
Gender	−0.02	0.03	0.03	0.04	−0.37 ***	−0.29 **	−0.31 ***	−0.16	−0.08	−0.09
Age	−0.03	−0.04	−0.03	−0.03	0.10	0.08	0.11 *	0.13 **	0.12 *	0.16 **
Education	0.09 *	0.09 *	0.09 *	0.09 *	0.24 *	0.24 *	0.17	0.09	0.08	0.01
Tenure	0.02	0.01	−0.00	−0.01	−0.03	−0.04	−0.05	−0.07	−0.08	−0.09
Salary	0.04	0.03	0.03	0.03	0.20 **	0.19 **	0.16 **	0.20 ***	0.19 ***	0.17 ***
Predictors										
SP		0.24 ***	0.23 ***	0.14 ***		0.47 ***	0.28 ***		0.38 ***	0.21 ***
Job crafting							0.79 ***			0.71 ***
Work stress			0.04 *	0.04 *						
SP × Work stress				0.06 ***						
*R* ^2^	0.01	0.09	0.10	0.11	0.05	0.12	0.23	0.06	0.12	0.23
Δ*R*^2^		0.08	0.01	0.02		0.06	0.12		0.06	0.11
*F-value*	1.37	69.15 ***	4.75 *	12.55 ***	8.73 ***	53.99 ***	117.01 ***	9.33 ***	55.69 ***	110.42 ***

Note. *N* = 778. All regression coefficients are unstandardized. * *p* < 0.05, ** *p* < 0.01, *** *p* < 0.001. SP = Self-oriented perfectionism. Models 1–4 predict job crafting. Model 1 includes the control variables. Model 2 adds self-oriented perfectionism. Model 3 adds work stress. Model 4 adds the interaction between self-oriented perfectionism and work stress. Models 5–7 predict radical creativity. Model 5 includes the control variables. Model 6 adds self-oriented perfectionism. Model 7 adds job crafting. Models 8–10 predict incremental creativity. Model 8 includes the control variables. Model 9 adds self-oriented perfectionism. Model 10 adds job crafting.

**Table 5:** The relationship between self-oriented perfectionism and job crafting under different levels of work stress.

Work Stress	Effect	*SE*	*t*-Value	*p*-Value	LLCI	ULCI
Low level (MEAN − 1SD)	0.14	0.04	3.56	0.00	0.06	0.22
Medium level (MEAN)	0.23	0.03	7.91	0.00	0.17	0.29
High level (MEAN + 1SD)	0.32	0.04	8.34	0.00	0.25	0.40

Note. *N* = 778. Bootstrap = 5000, 95% confidence interval.

**Table 6:** The moderating effect of work stress on the mediating effect of job crafting.

Work Stress	Effect	BootSE	BootLLCI	BootULCI
Self-oriented perfectionism → Job crafting → Radical creativity				
Low level (MEAN − 1SD)	0.08	0.03	0.03	0.14
High level (MEAN + 1SD)	0.18	0.03	0.13	0.24
Difference	0.10	0.03	0.04	0.16
Index	0.05	0.02	0.02	0.08
Self-oriented perfectionism → Job crafting → Incremental creativity				
Low level (MEAN − 1SD)	0.07	0.03	0.02	0.12
High level (MEAN + 1SD)	0.15	0.03	0.11	0.20
Difference	0.09	0.03	0.03	0.14
Index	0.04	0.01	0.02	0.07

Note. *N* = 778. Bootstrap = 5000, 95% confidence interval.

## Data Availability

The data sets generated during and/or analyzed during the current study are available from the corresponding author on reasonable request.

## Appendix A. Measurements and Items

### Appendix A.1. Self-Oriented Perfectionism

1. It makes me uneasy to see an error in my work.

2. One of my goals is to be perfect in everything I do.

3. I aim for perfection in my work.

4. I must work to my full potential at all times.

5. I must always be successful at school or work.

### Appendix A.2. Work Stress

1. My job is extremely stressful.

2. Many stressful things happen to me at work.

3. I feel a great deal of stress because of my job.

### Appendix A.3. Job Crafting

1. I try to develop my capabilities.

2. I try to develop myself professionally.

3. I try to learn new things at work.

4. I make sure that I use my capacities to the fullest.

5. I decide on my own how I do things.

6. I make sure that my work is mentally less intense.

7. I try to ensure that my work is emotionally less intense.

8. I manage my work so that I try to minimize contact with people whose problems affect me emotionally.

9. I organize my work so as to minimize contact with people whose expectations are unrealistic.

10. I try to ensure that I do not have to make many difficult decisions at work.

11. I organize my work in such a way to make sure that I do not have to concentrate for too long a period at once.

12. I ask my supervisor to coach me.

13. I ask whether my supervisor is satisfied with my work.

14. I look to my supervisor for inspiration.

15. I ask others for feedback on my job performance.

16. I ask colleagues for advice.

17. When an interesting project comes along, I offer myself proactively as project co-worker.

18. If there are new developments, I am one of the first to learn about them and try them out.

19. When there is not much to do at work, I see it as a chance to start new projects.

20. I regularly take on extra tasks even though I do not receive extra salary for them.

21. I try to make my work more challenging by examining the underlying relationships between aspects of my job.

### Appendix A.4. Radical Creativity

1. I am a good source of highly creative ideas.

2. I demonstrate originality in my educational and teaching work.

3. I often suggest radically new ways for education and teaching.

### Appendix A.5. Incremental Creativity

1. I use previously existing ideas or work in an appropriate new way.

2. I am very good at adapting existing ideas or teaching approaches.

3. I easily modify previously existing work processes to suit current needs.
